# Association of metformin use with fracture risk in type 2 diabetes: A systematic review and meta-analysis of observational studies

**DOI:** 10.3389/fendo.2022.1038603

**Published:** 2023-01-11

**Authors:** Yining Wang, Liming Yu, Zhiqiang Ye, Rui Lin, Antonia RuJia Sun, Lingna Liu, Jinsong Wei, Feifu Deng, Xiangxin Zhong, Liao Cui, Li Li, Yanzhi Liu

**Affiliations:** ^1^ Zhanjiang Key Laboratory of Orthopaedic Technology and Trauma Treatment, Zhanjiang Central Hospital, Guangdong Medical University, Zhanjiang, China; ^2^ Guangdong Provincial Key Laboratory for Research and Development of Natural Drug, School of Pharmacy, Guangdong Medical University, Zhanjiang, China; ^3^ Department of Stomatology, Affiliated Hospital of Guangdong Medical University, Zhanjiang, China; ^4^ Centre for Biomedical Technologies, Queensland University of Technology, Brisbane, Queensland, Australia; ^5^ Center for Translational Medicine Research and Development, Shenzhen Institute of Advanced Technology, Chinese Academy of Science, Shenzhen, Guangdong, China; ^6^ Marine Medical Research Institute of Zhanjiang, Zhanjiang, China; ^7^ Department of Orthopedics, Affiliated Hospital of Guangdong Medical University, Zhanjiang, China

**Keywords:** fracture, diabetes, metformin, bone, meta-analysis

## Abstract

**Aims:**

Increasing evidence suggests that metformin can affect bone metabolism beyond its hypoglycemic effects in diabetic patients. However, the effects of metformin on fracture risk in type 2 diabetes mellitus (T2DM) patients remain unclear. A systematic review and meta-analysis were performed in this study to evaluate the association between metformin application and fracture risk in T2DM patients based on previous studies published until June 2021.

**Methods:**

A systematic search was performed to collect publications on metformin application in T2DM patients based on PubMed, Embase, Cochran, and Web of Science databases. Meta-analysis was performed by using a random-effects model to estimate the summary relative risks (RRs) with 95% confidence intervals (CIs). Subgroup analyses based on cohort/case-control and ethnicity and sensitivity analyses were also performed.

**Results:**

Eleven studies were included in the meta-analysis. Results demonstrated metformin use was not significantly associated with a decreased risk of fracture (RR, 0.91; 95% CI, 0.81–1.02; I^2 =^ 96.8%). Moreover, metformin use also demonstrated similar results in subgroup analyses of seven cohort studies and four case-control studies, respectively (RR, 0.90; 95% CI, 0.76–1.07; I^2 =^ 98.0%; RR, 0.96; 96% CI, 0.89–1.03; I^2 =^ 53.7%). Sensitivity analysis revealed that there was no publication bias.

**Conclusion:**

There was no significant correlation between fracture risk and metformin application in T2DM patients. Due to a limited number of existing studies, further research is needed to make a definite conclusion for clinical consensus.

## 1 Introduction

Diabetes mellitus (DM) is one of the leading causes of mortality and reduced life expectancy (1, 2). The estimated global number of individuals diagnosed with DM has increased from 422 million in 2014 (3) to over 536.6 million currently, and it is projected to reach 783.2 million by 2045, accounting for 12.2% of 20-79 year-olds (4). Type 2 DM (T2DM) represents approximately 90%–95% of all DM cases (5, 6). Diabetes-related complication costs are substantial and have significantly increased the healthcare burden of diabetes patients (7, 8). The estimated global direct health cost of diabetes is projected to rise to $845 billion by 2045 (9). Previous studies have demonstrated an increased risk for fragility fractures as an important complication of T2DM (10–12).

In contrast to patients with type 1 diabetes, T2DM patients exhibited increased or normal bone mineral density in the clinic but with increased bone fragility and fracture risk (13–15). The pivotal causes of higher bone fragility in T2DM are strongly associated with the phenotype of abnormal osseous architecture (especially the increased cortical porosity), collagen disorganization, bone vasculopathy, increased bone marrow adiposity, and low bone turnover, which together contribute to impairments in bone material properties (16–18). Patients with T2DM who have suffered fractures are prone to frequent wound infections, resulting in delayed fracture healing and an increased risk of nonunion or pseudoarthropathy (19–21). Fractures in T2DM patients result in prolonged immobility and hospitalizations and lead to substantial morbidity and mortality. In addition to the direct effects of diabetes on bone fragility, current medical management of T2DM also substantially impacts bone health and fracture risk (22, 23). For instance, thiazolidinediones have been associated with an increased fracture risk (24, 25), whereas metformin administration has been shown to have a protective effect on the bone health of diabetic patients (24–26).

Metformin, a biguanide antidiabetic drug, is considered the standard initial treatment for T2DM patients. It affects several aging-related processes, including bone deterioration, by suppressing cellular senescence and chronic inflammation and promoting autophagy (27, 28). Previous studies demonstrated that metformin directly promoted osteoblastic differentiation of different kinds of stem cells (including umbilical cord mesenchymal stem cells (29), adipose-derived stem cells (30), dental pulp stem cells (31), and bone marrow derived mesenchymal stem cells (32), enhanced the anabolic action of the bone (including beneficial effects on bone microarchitecture, bone mineral density, and bone turnover markers), and improved bone quality in patients with T2DM (33–36). Furthermore, a previous report suggested a potential benefit of metformin in contributing to decreased bone cancer risk in T2DM patients (37).

Nonetheless, whether metformin can reduce the risk of fractures remains unconfirmed and controversial. Previous studies have reported no significant correlation between fracture risk and metformin application (38) and no significant effects of metformin on bone marrow density (BMD) (39, 40). However, another investigation showed that 10 μg/mL of metformin might partially suppress the mineralization of osteoblasts (41). Borges et al. found that the effects of metformin monotherapy showed only small but not significant increases in lumbar spine BMD at all time points from baseline to week 80 in T2DM patients (42). Therefore, the effect of metformin on bone metabolism and whether metformin medication reduces the risk of fracture in patients with T2DM needs further evaluation.

In this study, to determine whether metformin treatment could reduce fracture risk in T2DM, a comprehensive meta-analysis was performed on the fracture risk of T2DM patients receiving metformin administration; it included all previous reports up to June 2021.

## 2 Methods

### 2.1 Search strategy

This meta-analysis was performed according to the Preferred Reporting Items for Systematic reviews and Meta-Analyses (PRISMA) statement guideline for systematic reviews and meta-analyses (43, 44), and it was registered with PROSPERO (No. CRD42022344967). We searched for studies on the fracture risk of diabetic patients with metformin administration published until June 2021 by using PubMed, Embase, Cochran library, and Web of Science databases. The following keywords were used for publication collection: (“Metformin” OR “dimethylbiguanide” OR “metformin HCI”) AND (“bone” OR “bone fracture” OR “fracture” OR “osteoporotic fracture” OR “broken bone” OR “bone mineral density” OR “BMD” OR “bone mass density” OR “osteoporosis” OR “bone health” OR “bone quality”).

### 2.2 Inclusion criteria

Each title and abstract were reviewed to identify relevant papers. Full texts of the articles were reviewed if the abstract was deemed potentially relevant. Studies that met the following criteria were eligible for inclusion:

(1) observational studies where metformin was the exposure variable and fractures were the main outcome variable or one of the outcome variables.
(2) T2DM participants aged ≥18 years.
(3) The odds ratio (OR), risk ratio (RR), and hazard ratio (HR) were reported as the effect size (ES).

### 2.3 Exclusion criteria

The exclusion criteria excluded studies that:

(1) Related to other drugs in combination treatment with metformin.
(2) Excluded placebo diabetic control.
(3) Included Type 1 diabetes patients.
(4) Included single gender.

**Figure 1:**
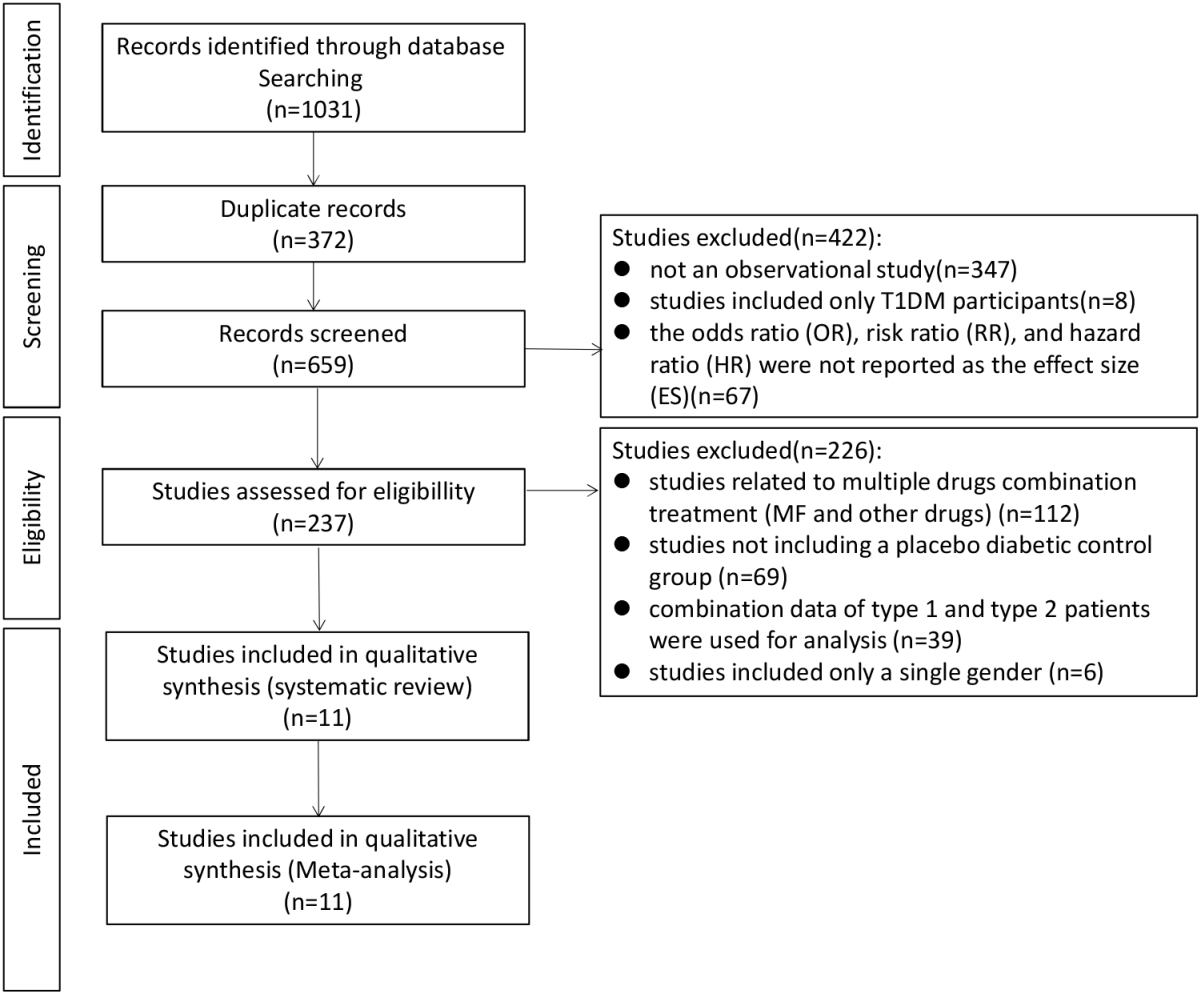
Flowchart of the study selection process.

A total of 1,031 publications were identified with the search strategy. Then, these studies were independently screened according to the inclusion and exclusion criteria. A total of 11 studies were eligible and included in the meta-analysis. (**Figure 1**)

### 2.4 Data extraction

Two authors (WYN and YLM) independently conducted study screening and data extraction from the eligible literature. When disputes were encountered, they were resolved through discussion or assisted by the main investigator (LYZ). The following data were collected from all included studies: first author’s surname, publication year, study design, country, follow-up duration, mean age or age range of participants, gender, sample size, number of cases, outcome variables and fracture assessment method, the adjusted ORs, RRs, or HRs, and the corresponding 95% confidence intervals (CIs) (**Table 1**). The Newcastle-Ottawa Scale (NOS) quality assessment was used to evaluate the studies.

**Table 1:** Characteristic table of studies on the relationship between MF use and fracture risk in diabetic patients.

First author (year)	Country	Duration of follow-up (years)	Design	Mean age	Samples sizes	Cases	Adjusted OR, RR, or HR (95% CI)	Nos	Fracture types
Tseng CH (2021) (37)	Taiwan	5.3	Cohort	62	29,222	977	0.62 (0.55,0.71)	9	vertebral fracture
Tak Kyu Oh (2020) (38)	South Korea	5	Cohort	60	37378	972	1.00 (0.86,1.16)	7	Hip fracture
Starup-Linde J (45)	Denmark	5.5	Cohort	Cases: 58 Controls:73	180,073	20,557	0.73 (0.71,0.76)	9	Any fracture, vertebral fracture, forearm fracture, and osteoporotic fracture
Wallander M (46)	Sweden	1.3	Cohort	Cases: 79 Controls:81	79,159	2394	1.05 (0.96,1.14)	9	Hip fracture
Hung YC (47)	Taiwan	3.9	Cohort	70	7,761	367	1.02 (0.83,1.26)	9	Hip fracture
Majumdar (48)	USA	2.2	Cohort	52	72,738	741	1.00 (0.80,1.20)	9	Osteoporotic fracture
Colhoun (49)	Scotland	9	Cohort	65	173,113	2,433	1.02 (1.00,1.05)	9	Hip fracture
Charlier (50)	UK	23	Case-control	71	28617	5692	1.00 (0.95,1.05)	8	Low-trauma fracture (non-vertebral fractures of the proximal and distal upper and lower extremities, ribs and thorax, hip and foot)
Vavanikunnel J (51)	UK	21	Case-control	72	27124	5366	0.96 (0.91,1.01)	8	Low-trauma fracture (non-vertebral fractures of the proximal and distal upper and lower extremities, ribs and thorax, hip, and foot)
Puar TH (52)	Singapore	5	Case-control	Cases: 77 Controls:76	1,116	558	0.73 (0.57,0.94)	8	Hip fracture
Monami M(53)	Italy	4.1	Nested Case-control	Cases: 69 Controls:68	332	83	0.94 (0.54,1.65)	8	Total fracture

Mean age only displayed integers, ignoring numbers after the decimal point. OR, odds ratio; RR, risk ratio; HR, hazard ratio; CI, confidence interval; Nos, Newcastle-Ottawa scale.

### 2.5 Statistical analysis

Stata software was used for meta-analysis (Stata, version 16, College Station, TX, USA). All reported ORs, RRs, HRs, and the 95% CIs for fracture risk were used to calculate the logarithmic RR and its standard error (SE). A random effects model was used to estimate the summary relative risks with 95% CIs. Q and I^2^ tests were performed to analyze the homogeneity of the included studies. For the Q test, statistical significance was set at p ≤ 0.05; for I^2^ statistics, the following critical points were specified to define the degree of heterogeneity: <25% (low heterogeneity), 25%–50% (moderate heterogeneity), 50%–75% (high heterogeneity), and >75% (severe heterogeneity).

The subgroup analyses of cohort, case-control, and ethnicity were respectively performed on the included studies. In addition, sensitivity analysis was used to investigate the extent to which inferences might depend on a particular study or research group. Visual inspection of the funnel chart was used to assess publication bias. A formal statistical assessment of funnel plot asymmetry was performed using Egger’s regression asymmetry test. *P* values of < 0.05 were considered statistically significant.

## 3 Results

### 3.1 Study characteristics

Among the 1,031 retrieved papers, 11 related to the application of metformin and the risk of fracture in T2DM patients and were included in this meta-analysis according to the inclusion/exclusion criteria (**Table 1**). Among the 11 studies, there were seven cohort studies (38, 45–49, 54), and four case-control studies (50–53). All studies included both genders (**Figure 1**).

These studies collectively included 635,945 participants and were published between 2008 and 2021; they were conducted in various regions, including one study each in the United States (48), Denmark (45), South Korea (38), Singapore (52), Italy (53), and Sweden (46), two in Taiwan (47, 54), and three in the United Kingdom (49–51).

Regarding the types of fractures, five studies included fractures at multiple sites, such as fractures of the proximal and distal upper and lower extremities, ribs and thorax, hip, and foot (45, 48, 50, 51, 53), two studies included vertebral fracture (45, 54), six studies included hip fractures (38, 45–47, 49, 52), and three studies included osteoporotic fracture (45, 46, 48).

### 3.2 Results of the meta-analysis

There were seven cohort studies and four case-control studies involved in the meta-analysis. Four studies revealed that metformin treatment reduced fracture risk, seven studies demonstrated that metformin had no significant effect associated with fracture risk, and no studies showed that metformin increased fracture risk.

**Figure 2:**
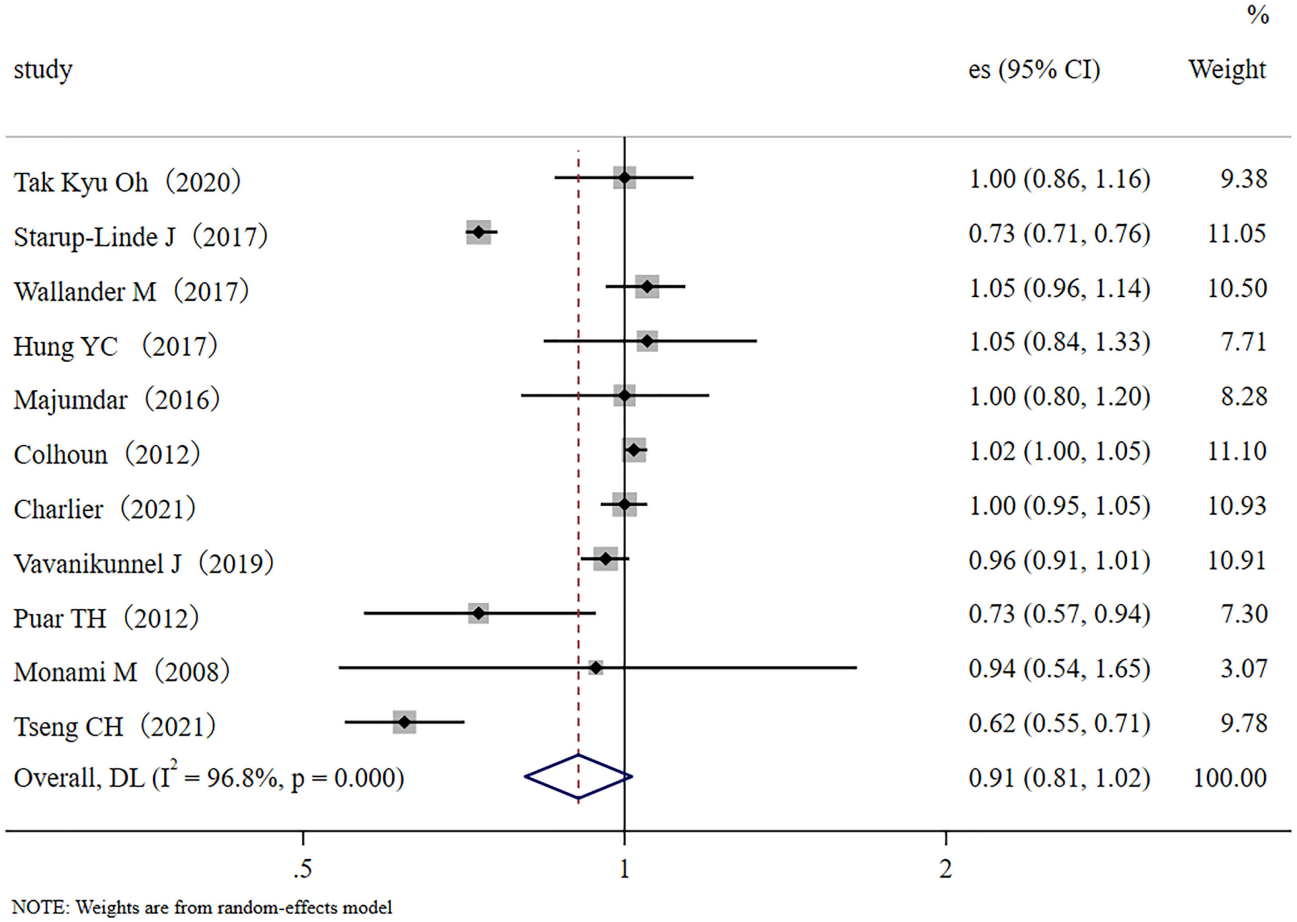
Forest plot of the 11 studies that examined the association between metformin application and fracture risk in T2DM patients; (Study: author and year of publication; es, effect size; 95% CI, 95% confidence interval).

In this meta-analysis, we had 11 effect sizes obtained from 11 studies. The meta-analysis results are shown as forest plots (**Figure 2**). Results demonstrated that metformin administration was not significantly associated with a decrease in the fracture rate of diabetic patients (RR, 0.91; 95% CI, 0.81–1.02). Significantly high between-study heterogeneity was found (I^2^ = 96.8%, *p* < 0.001).

In the sensitivity analysis, no single study significantly influenced the findings. No evidence of publication bias was found in this meta-analysis with Egger’s test evaluation (*p* = 0.99).

## 4 Discussion

The occurrence of fractures is closely related to low BMD and osteoporosis development (55, 56). Previous studies have demonstrated that BMD significantly decreased in patients with T1DM, leading to an increased risk of fractures (57). Although T2DM patients showed bone formation suppression, microarchitecture deterioration, and microvascular complications in the bone (58), unlike T1DM patients, T2DM patients might not demonstrate significant BMD decline (59). Since T2DM accounts for more than 90%–95% of all diabetes cases, the factors associated with type 2 diabetic fractures attract a lot of concerns. The effects of T2DM on bone are multifactorial, including hyperglycemia (60), insulin imbalance (61), obesity (62), and medications (63). Among several factors that might influence the risk of fracture, much attention has been given to glucose-lowering medications, for example, metformin.

In this study, we performed a meta-analysis and a series of sub-group meta-analyses to examine the association between metformin use and the risk of fracture in T2DM patients. We found no significant association between metformin use and fracture risk. Due to only a small number of studies (n=11) being included in this study, investigation of fractures in specific sites was not possible; therefore, our study used the same strategy as that of a previous report (64) that focused on the association of metformin use and fracture risk from any sites with no focus on a specific site.

**Figure 3:**
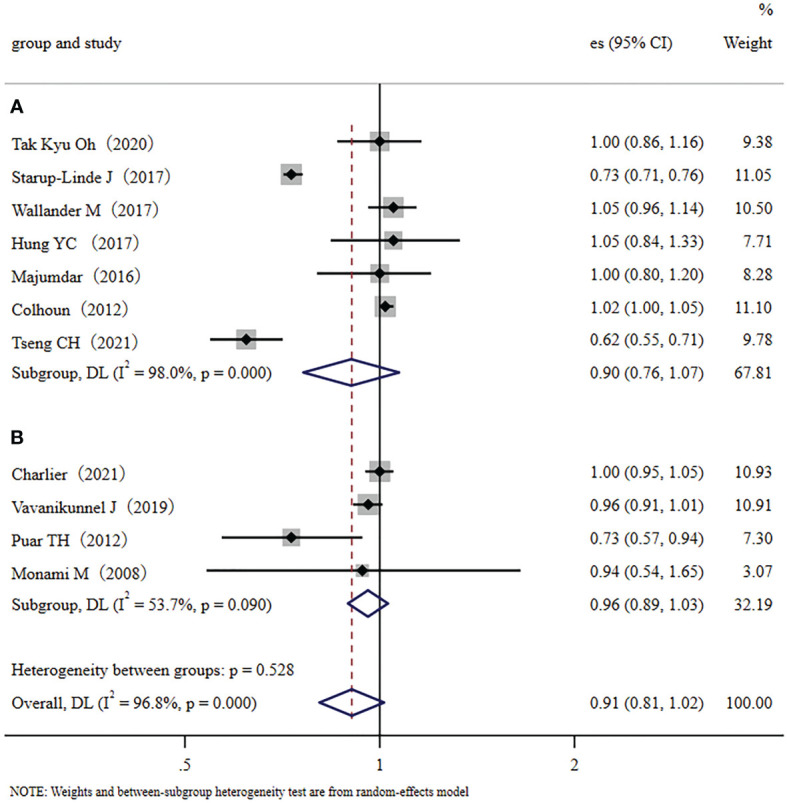
The cohort/case-control subgroup analysis: **(A)**: Forest plot of the included Cohort studies that examined the association between metformin application and fracture risk in type 2 diabetic patients; **(B)**: Forest plot of the included Case-control studies that examined the association between metformin application and fracture risk in type 2 diabetic patients. (Study: author and year of publication; es, effect size; 95% CI, 95% confidence interval; Weight, weight).

**Figure 4:**
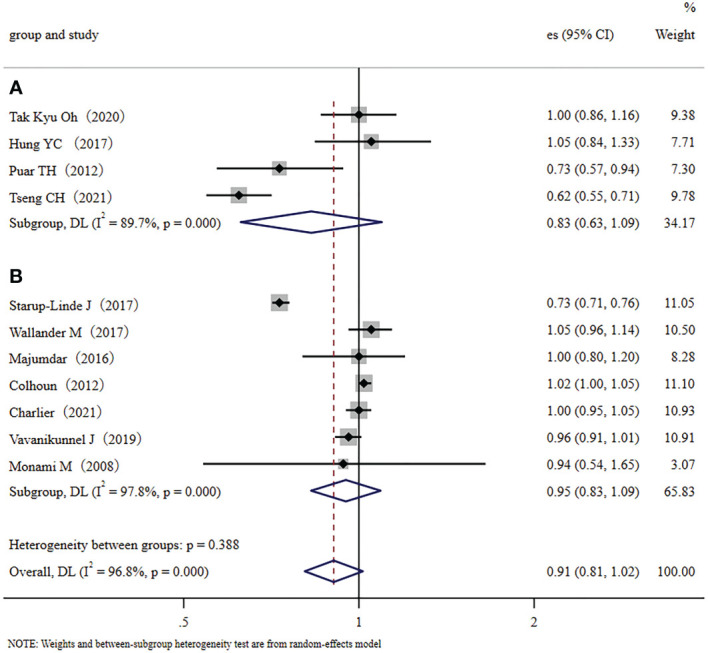
The ethnic subgroup analysis: **(A)**: Forest plot of the four studies that were performed in Asia (major population: Far-Eastern origins); **(B)**: Forest plot of the seven studies that were performed in Europe/the United States (major population: European origins); Study: author and year of publication; es, effect size; 95% CI, 95% confidence interval; Weight, weight).

We conducted a subgroup meta-analysis that only included the seven cohort studies to demonstrate multiple validations of our conclusion. The results showed that metformin administration was not closely related to a decreased fracture risk in diabetic patients (RR, 0.90; 95% CI, 0.76–1.07). Inter-study heterogeneity was significant; I^2 =^ 98.0%, *p* < 0.001 (**Figure 3A**). We also conducted a subgroup meta-analysis that only included the four case-control studies. Like the cohort studies, the case-control studies also demonstrated that metformin administration was not closely related to a decreased fracture risk in diabetic patients (RR, 0.96; 95% CI, 0.89–1.03), but heterogeneity was not significant, I^2 =^ 53.7%, *p*=0.090 (**Figure 3B**). In addition, ethnicity was used as a categorical variable for subgroup analysis. Based on the eleven collected studies, four studies were performed in Asian countries (traditional major population mainly of Far-Eastern origins), and seven were performed in Europe/the United States of America (traditional major population mainly of European origins). Therefore, we used the Far-Eastern origins/the European origins as the ethnic category for sub-group analysis. Results demonstrated that metformin administration was not closely related to a decreased fracture risk in diabetic people of Far-Eastern origins (RR 0.83, 95% CI, 0.63–1.09) (**Figure 4A**). The subgroup analysis in the diabetic people of European origins similarly demonstrated that metformin administration was not closely related to a decreased fracture risk (RR 0.95, 95% CI, 0.83–1.09) (**Figure 4B**). Overall, the subgroup meta-analyses demonstrated that different subgroup analyses support the same conclusion.

After subgroup analyses, we conducted a bias analysis on the quality of the included studies, and STATA was used to prepare a funnel chart for the 11 included studies. Funnel plot analysis showed that five studies might significantly affect the overall heterogeneity of the analysis (**Figure S1**). We excluded four of the five studies that may have affected the overall heterogeneity of this analysis (Starup-Linde et al., 2017 (45), Colhoun et al., 2012 (49), Puar et al., 2012 (52), and Tseng et al., 2021 (54)) and the remaining seven studies were used for further meta-analysis (**Figure S2**). The results further demonstrated that metformin administration and the fracture risk of diabetic patients were not significantly inversely related (RR, 0.99; 95% CI, 0.96–1.02). The heterogeneity between studies was I^2 =^ 0.0%, which demonstrated that the high heterogeneity across all 11 studies did not significantly affect the results in this meta-analysis. Therefore, the conclusion of this meta-analysis was robust and credible. The Egger’s test result was *p*=0.9301 and *p*>0.05, which indicated that the meta-analysis has no publication bias. A sensitivity analysis was used to test the stability of the effect size (ES) estimates (**Figure S3**). Overall, the data of these analyses all suggested that metformin use is not significantly associated with a decreased risk of fractures in T2DM patients.

Several studies demonstrated that metformin treatment was associated with significant low bone fracture risks in patients with diabetes (65, 66). The treatment of T2DM patients and osteoporosis with metformin and dietary intervention could decrease blood glucose levels, increase bone density, and alleviate osteoporosis (67–70). A recent study also reported that metformin use was associated with a lower risk of osteoporosis/vertebral fracture in T2DM patients (37). A prior systematic review and meta-analysis suggested that metformin use was inversely associated with the risk of fracture in diabetes (RR, 0.82; 95% CI, 0.72–0.93; n=7; I^2 =^ 22.4%; *p*=0.259) (64). Another meta-analysis demonstrated that the use of metformin appears to decrease the fracture risk (RR, 0.86; 95% CI, 0.75–0.99; I^2 =^ 95.2%; *p <*0.001). The reduced fracture risk with metformin could be related to metformin prescriptions that typically start in the early stages of type 2 diabetes mellitus (63). Furthermore, metformin has several relevant contraindications, including renal insufficiency, severe liver disease, and heart failure. A lower comorbidity may contribute to the influence of metformin on the lower incidence of bone fractures (53).

This study found that metformin use is not significantly associated with a decreased risk of fractures in T2DM patients. Our results are inconsistent with previous studies including type 1 and type 2 patients and single-gender data. Our study focused on T2DM patients and only included studies that examined both genders. A previous study by Wallander et al. (46), demonstrated that women with T2DM-oral medication had an increased risk of hip fracture compared to men. Therefore, we excluded studies that reported on single-gender involvement. According to the data we collected in the 11 studies, four studies revealed that metformin treatment could reduce fracture risk (45, 50, 52, 54), and another seven demonstrated that metformin had no significant effect associated with fracture risk (38, 46–48, 52). None of the studies in this review showed that metformin increased fracture risk. The differing results between studies may be due to variations in metformin dose and duration. However, we noted that all the included observational studies did not reveal the specific metformin dose, which makes it difficult to interpret and analyze the underlying reason. To model the univariate effects of metformin, the 11 included studies selected individuals who could be stratified based on cumulative exposure to metformin. For example, current metformin users were defined by Charlier et al. as participants with their last prescription ≤ 60 days prior to the index date (50), whereas Colhoun et al. included metformin users with a cumulative exposure of 1 year (49). Therefore, in this meta-analysis, we focused on T2DM patients with current metformin duration (at least>30 days) as the outcome and ignored the dose. We summarized the relevant information on metformin administration reported in the 11 included studies (**Table S1**). The data on ever-exposure to metformin was not used in this study, as we assumed that there were no legacy or carry-over effects from remote exposure to any antidiabetic drugs. Colhoun et al. considered that cumulative metformin exposure does not depend on the events in the unexposed and, therefore, cannot be affected by allocation bias (49). We used cumulative metformin exposure in this study was consistent with them. Therefore, the data from current cumulative exposure to metformin was considered more accurate than data from ever exposure to metformin. In addition, though several studies have various sub-group settings, they may not provide comprehensive information for analysis. Therefore, we preferred to use the total integrated data for the present analysis. For example, in a study by Charlier et al. (50), current metformin exposure was divided into three categories: ①HbA1c ≤ 7.0%, ②HbA1c>7.0% and ≤ 8.0%, and ③HbA1c>8.0%. Our study only collected the general comprehensive data for analysis, independently based on patients’ HbA1c levels. The results demonstrated that metformin treatment was not significantly associated with fracture risk. However, we noticed that HbA1 level is an important parameter in metformin use that may significantly affect fracture risk. Patients with current metformin use that controlled the HbA1 levels at the range of ≤7.0% and >7.0% but ≤8.0% demonstrated a significantly reduced risk of fractures (aOR, 0.89; 95% CI, 0.83–0.96, and 0.81; 95% CI, 0.73–0.90, respectively). The results suggested that proper blood sugar management by metformin may help to decrease fracture risk. However, Hung et al. demonstrated that severe hypoglycemia in T2DM patients significantly increases the risk of falls and the cumulative incidence of hip fracture (47). The study by Puar et al. also suggested a greater risk of falls in older adults with tight glycemic control (HbA1c<7%) (52). If metformin administration significantly contributes to severe hypoglycemia, then the fall risk may increase and decrease the beneficial effects of metformin on bone. Wallander et al. suggested that metformin administration was independently associated with an increased risk of non-skeletal fall injury (46).

In summary, our data demonstrated that metformin treatment was not significantly associated with the risk of fracture, and our results are independent of patients’ HbA1c levels/glycemic control levels. When the data with HbA1c control is considered for analysis, for example, If the data of HbA1c ≤ 7.0% (OR, 0.89; 95% CI, 0.83–0.96) (Charlier, 2021) (50) was used for our meta-analysis, it shifted the overall estimate (OR, 0.87; 95% CI, 0.77–0.97) and the results demonstrated that metformin treatment was related to a decreased fracture risk in T2DM patients (**Figure S4**).

Metformin is often prescribed in combination with other antidiabetic medications. The possible effect of interaction between metformin with other antidiabetic medications may also affect bone fracture risk. A previous study investigated the effect of metformin relative to placebo in combination with insulin analogs (Metformin + Insulin vs. Placebo + Insulin) on bone markers P1NP Procollagen type 1 N-terminal propeptide (P1NP) and C-terminal telopeptide of type I collagen (CTX) in patients with T2DM (71). The levels of bone formation marker P1NP and bone resorption marker CTX increased significantly in both groups. However, the Metformin+Insulin combination increased P1NP less than the Placebo+Insulin combination. There was no statistical difference in CTX between groups. There were no adverse effects on bone or muscle when metformin was used in combination with sitagliptin (72). The current use of metformin plus SGLT-2 inhibitor compared to the current use of metformin plus DPP-4 inhibitor was not associated with fractures in patients with type 2 diabetes (73). SGLT2 inhibitors + metformin combination treatment do not affect fracture risk compared to GLP-1 receptor agonists + metformin combination (74). SGLT2 and metformin combination therapy did not influence fracture risk compared with metformin monotherapy or other medications in patients with T2DM (75). Low-dose combination therapy with rosiglitazone and metformin was highly effective in preventing type 2 diabetes in patients with impaired glucose tolerance, with little effect on the clinically relevant adverse events of these two drugs (76). Another previous study demonstrated that metformin combined with sulfonylurea, meglitinide, acarbose, pioglitazone, immunosuppressants, or estrogen (women only) for diabetes management, all revealed a significant association with lower fracture risk. However, metformin combined with insulin or rosiglitazone for diabetes management did not show a decreased fracture risk. Significant interactions between metformin, insulin, sulfonylurea, and pioglitazone were found (*p*-values for interaction<0.05). The protective effect of metformin was not significant in insulin-treated patients, while metformin revealed greater beneficial effects in sulfonylurea or pioglitazone-treated patients (28). The possible effect of the interaction of metformin with other antidiabetic medications on bone fracture protection still needs more direct evidence to show specific indications clearly.

This meta-analysis has some limitations. There was significant heterogeneity between the 11 included studies. The reason may be due to differences in the sample sizes of the included studies. For instance, the study of Starup-Linde et al., 2017 (45) included the most fracture cases (20,557), while Monami et al., 2008 (53) included only 83 cases. The significant case differences in the sample size may have contributed to the high heterogeneity (77). Additionally, study differences in the quality, design, and country and continent of origin may have also contributed to the high heterogeneity. The high heterogeneity may have been caused by the difference in the strength of the correlation between the studies rather than the difference in the direction of the correlation (78). Further, the number of studies (n=11) included in this meta-analysis was limited, and it is expected that more sufficient samples and high-quality clinical data will be available in the future. Studies with a larger sample size will provide more accurate evidence to support metformin administration and its role in fracture risk in diabetic patients. In this study, we only focus on the association of metformin therapy and fracture risk. However, T2DM patients commonly use multiple medications for hyperglycemia management. The potential interaction effects between metformin and other antidiabetic medication on bone fracture protection also need more direct evidence to clearly show specific indication.

## 5 Conclusion

In this systematic review and meta-analysis, we found that metformin administration was not significantly correlated with a decreased fracture risk in T2DM patients. These results were independent of patients’ HbA1c levels and glycemic control levels. Due to the limited number of studies included in this meta-analysis, further investigations are needed to make stronger conclusions for clinical consensus.

## Data availability statement

The original contributions presented in the study are included in the article/**Supplementary Material**. Further inquiries can be directed to the corresponding authors.

## Author contributions

Study design/manuscript preparation: YL, LL, and LC. Original draft preparation/Meta analysis: YW and LY. Sub-Group Meta analysis and validation: ZY and RL. Review and editing: AS, LL, and JW. Conduct data/Publication collection/Analysis: FD, and XZ. Funding acquisition: YL, LL, and LC. All authors have read and agreed to the published version of the manuscript. All authors contributed to the article and approved the submitted version.
