# Supplementary material for: Association of metformin use with fracture risk in type 2 diabetes: A systematic review and meta-analysis of observational studies
Source: Front Endocrinol (Lausanne). 2023 Jan 11;13:1038603. doi: 10.3389/fendo.2022.1038603 (PMC9874692; doi:10.3389/fendo.2022.1038603)
Supplement: Supplementary file 1 [file DataSheet_1.docx]

#### Association of metformin use with fracture risk in type 2 diabetes: a systematic review and meta-analysis of observational studies

#### Supplementary materials


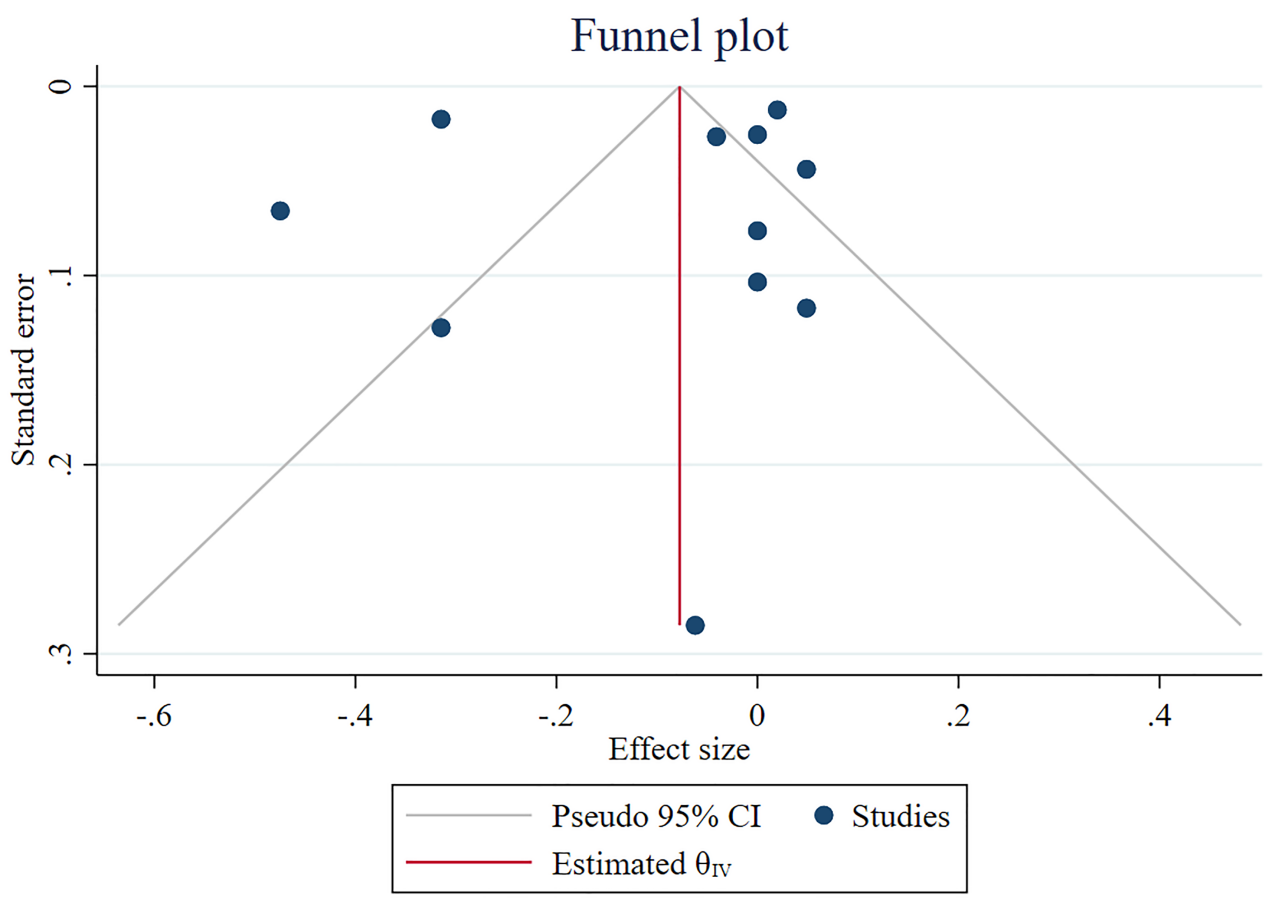


**Figure S1** Funnel chart of included 11 studies that examined the association between MF application and fracture risk in type 2 diabetic patients.


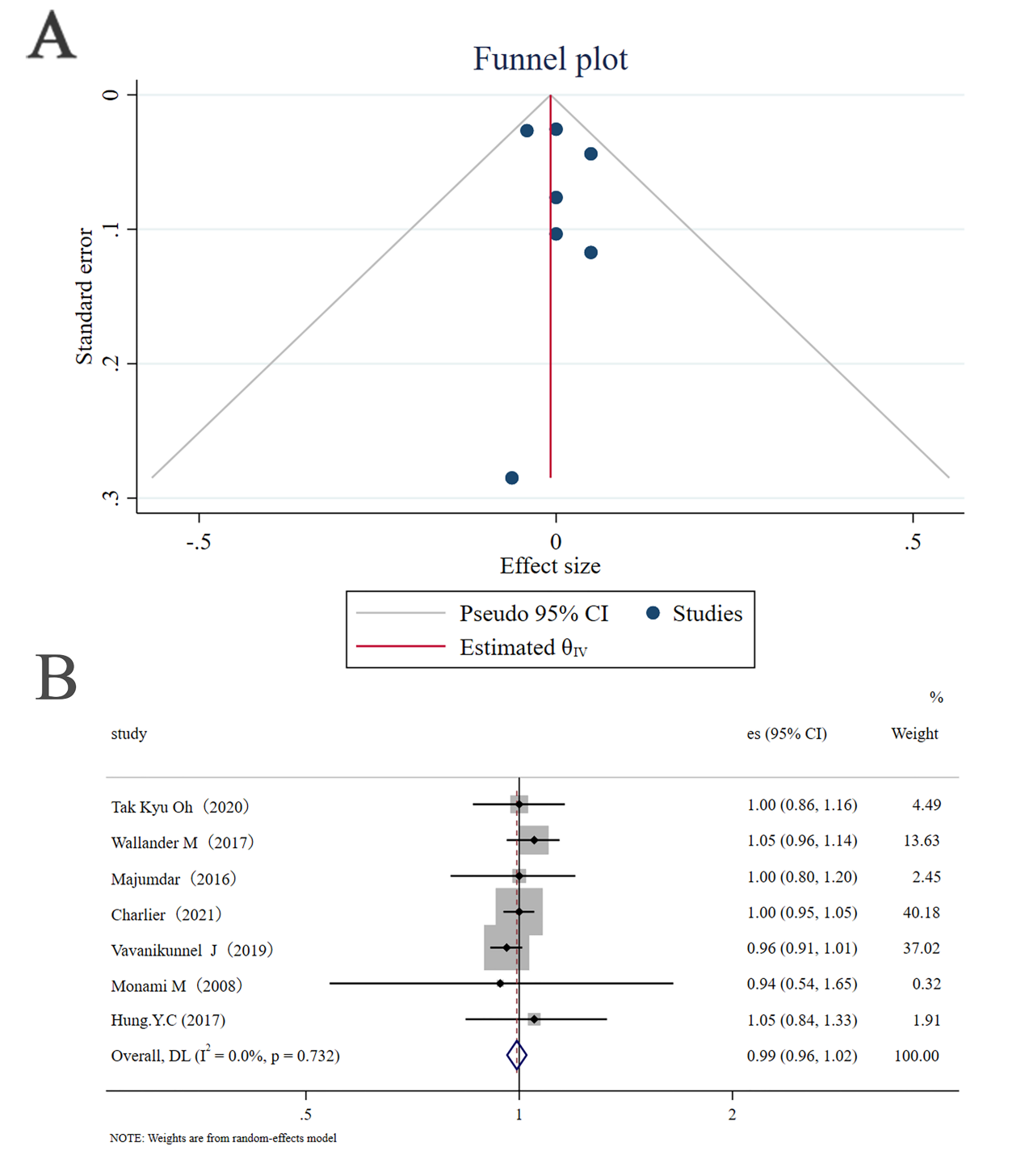


**Figure S2** A: Funnel chart of included 7 studies that examined the association between MF application and fracture risk in diabetic patients; B: Funnel chart of 7 studies the association between MF use and fracture risk in diabetic patients. (Study: author and year of publication; es: effect size; 95% CI: 95% confidence interval; Weight: weight)


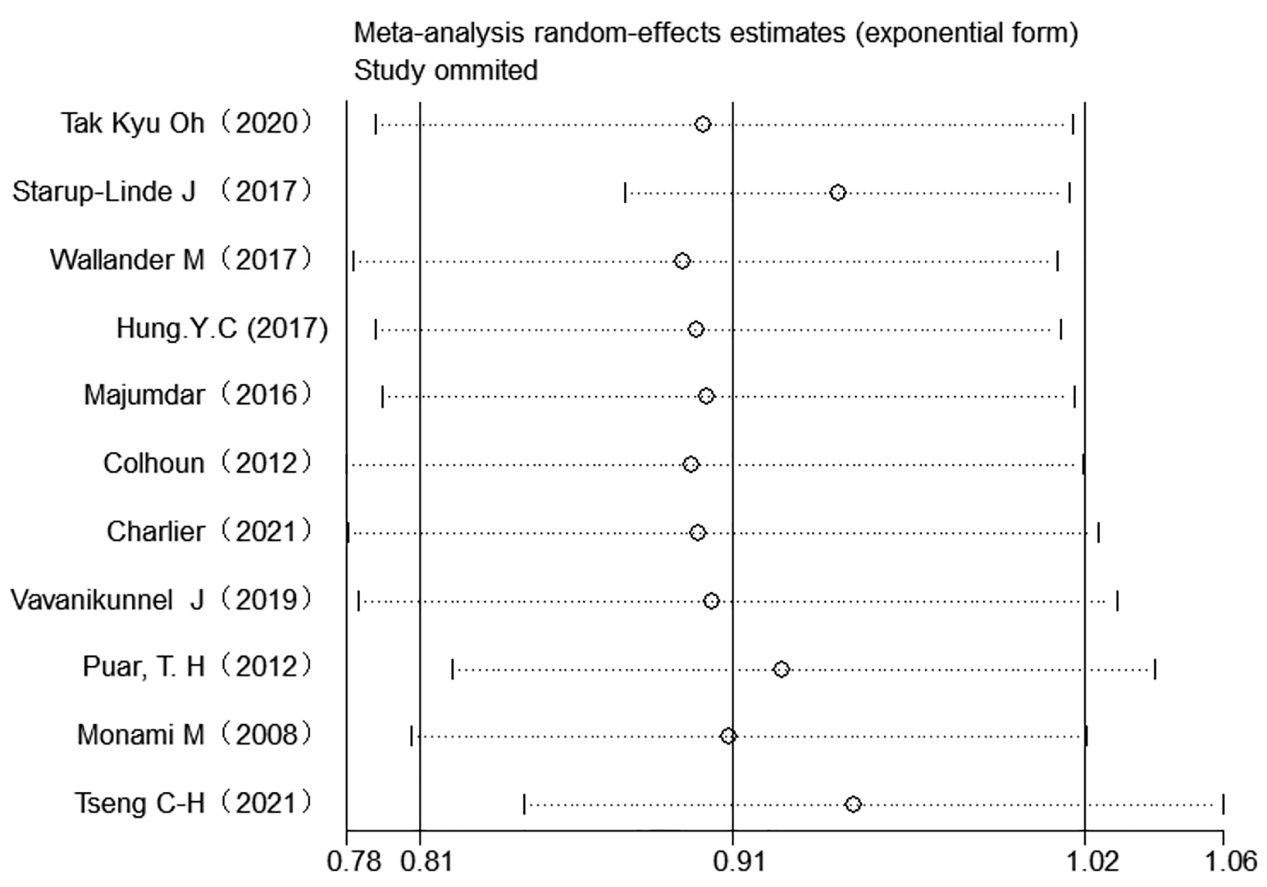


**Figure S3** Sensitivity analysis of included studies that examined the association between the use of MF and the risk of fracture in diabetic patients from 11 studies.


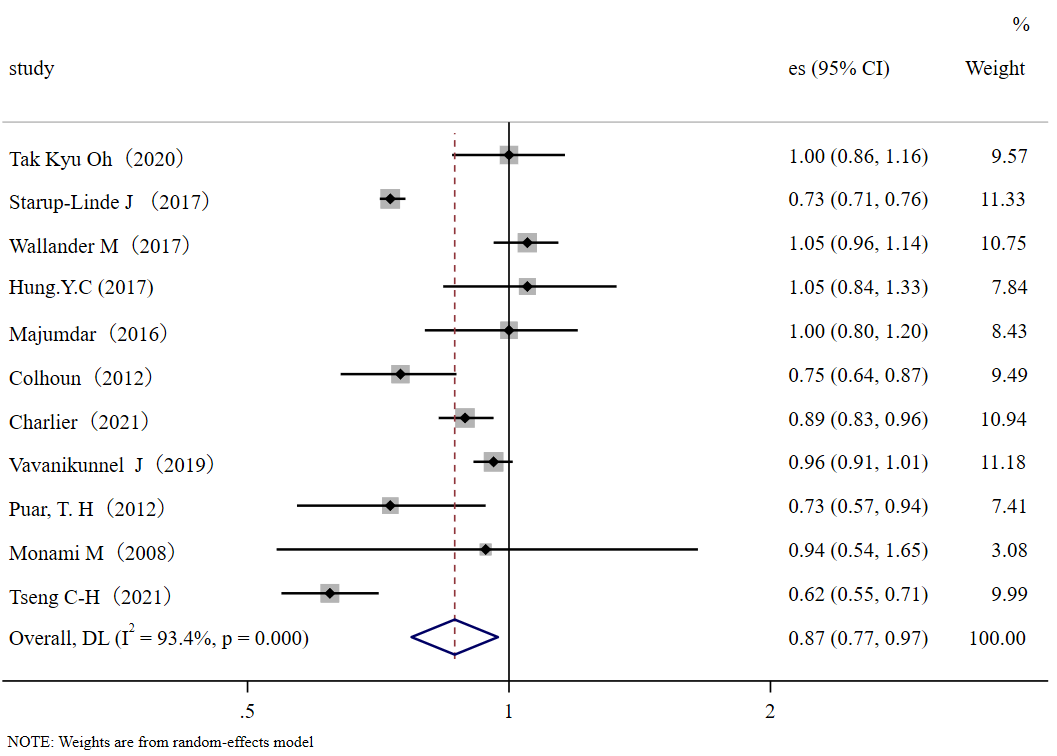


**Figure S4** Forest plot of the 11 studies that examined the association between MF application and fracture risk in T2DM patients; included the data with HbA1c control from the study of Charlier (2021) (HbA1c ≤7.0% (OR 0.89，95%CI 0.83–0.96) for analysis

**Table S1** Characteristics of Metformin use information in the included studies

| **Studies** | **Metformin dose** | **Focus** | **OR (95% CI)** | **Metformin-duration** | **Results** | **Sup-group results** |
| --- | --- | --- | --- | --- | --- | --- |
| ⑴ Sarah Charlier (2021) | No specific dose report, patients received MF dose based on NICE guidelines | Metformin use;  Fracture; | 1.00,  (95% CI 0.95–1.05) | >60 days  cumulative  exposure  (Time interval 60 days) | Independently of their HbA1c levels,  metformin was not materially associated with the risk of fracture | Current users of metformin (last prescription ≤60 days prior to the index date)  with HbA1c levels<8.0% had a decreased risk of fractures compared with non-users of any antidiabetic medication   - HbA1c ≤7.0%   OR 0.89, (95% CI, 0.83-0.96)   - HbA1c >7.0%-≤8.0%   OR 0.81, (95% CI, 0.73-0.90)   - HbA1c >8.0%   OR 0.95 (0.81-1.10) |
| ⑵ Chin-Hsiao Tseng (2021) | No specific dose report, cumulative duration of metformin therapy was used  as an indicator of dose–response relationship. | Current use of metformin;  primary outcome as vertebral fracture only diagnosed at outpatient clinics or during hospitalization | 0.622, (95% CI 0.547–0.708) | > 2 years | Current metformin  therapy > 2 years was consistently associated with a lower risk of vertebral fracture | Current metformin therapy > 2 years was consistently associated with a lower risk of vertebral fracture without a prior diagnosis of osteoporosis diagnosed at outpatient clinics or during hospitalization  OR 0.659, (95% CI, 0.564-0.770) |
| ⑶ Colhoun, H (2012) | No specific dose report, the cumulative exposure was calculated as years of exposure  (ignoring dose) to a model in which the cumulative exposure was calculated as the sum of daily doses | Current use of metformin; occurrence of hip fracture | 1.02,  (95% CI 1.00–1.05) | cumulative  exposure 1 year | No association of hip fracture with cumulative exposure to metformin | Ever metformin therapy was consistently associated with a lower risk of hip fracture |
| ⑷ Hung, Y. C (2017) | No specific dose report | Metformin use； hip fracture | 1.05 (95% CI 0.84–1.33) | three consecutive  months or longer use during the follow-up period | metformin use was  not significantly associated with the risk of hip fracture | N/A |
| ⑸ Majumdar, S. R (2016) | No specific dose report | Metformin use;  Major Osteoporotic Fractures (defined as any nontraumatic fragility fracture of the forearm, humerus, vertebrae, or hip) | 1.0 (95% CI 0.8–1.2) | Within each 90-day window of follow-up | There was no independent association between  current use of metformin and fracture | N/A |
| ⑹ Monami, M(2008) | No specific dose report | Fractures;  Metformin use | 0.94 (95% CI 0.54–1.65) | Exposure of > 1 year to Metformin treatments | No association  was observed between treatment with metformin and incident bone fractures in type 2 diabetic patients | N/A |
| ⑺ Puar, T. H (2012) | No specific dose report | Metformin use; hip fracture | 0.73 (95% CI 0.57–0.94) | Exposure of > 3 months to Metformin treatments | Use of metformin was protective for hip fractures. | N/A |
| ⑻ Starup-Linde, J (2017) | No specific dose report | Fractures;  Metformin use | 0.73(95% CI 0.71–0.76) | Exposure of > 30 days to Metformin treatments | Current use of metformin was associated with a decreased risk of any fracture | Hip fracture  Exposure of Metformin   - Ever used   0.45(95% CI 0.43,0.49)   - Use current 30 days   0.71(95% CI 0.66,0.77)   - Use current 90 days   0.61(95% CI 0.57,0.65)   - Use current six months   0.57(95% CI 0.53,0.61) |
| ⑼ Tak Kyu Oh (2020) | No specific dose report | Metformin therapy,  hip fracture | 1.00, (95% CI: 0.86–1.16) P=0.985 | the duration of exposure to metformin at least 1-year. | the exposure  to metformin was not significantly associated with the development of hip fracture compared to the control | Multivariable time-dependent Cox regression analysis, Metformin 0.78 (95% CI, 0.36, 1.69) P=0.525 |
| ⑽ Vavanikunnel, J (2019) | No specific dose report | current Metformin use;  Frature | 0.96 (95% CI: 0.91–1.01) | >60 days | metformin was not associated with the risk of  fracture in the patients with T2DM | N/A |
| ⑾ Wallander, M (2017) | No specific dose report | Metformin use;  hip fractures; | 1.05 (95% CI: 0.96–1.14) | A median follow-up time of 1.3 years (interquartile range 0.6–2.3) | Metformin use was  not associated with an increased risk of hip fracture . | N/A |

**Table S2** The Newcastle Ottawa scale for cohort study

| **Study** | **Selection** | | | | | **Comparability** | **Outcome** | | | **Total stars** |
| --- | --- | --- | --- | --- | --- | --- | --- | --- | --- | --- |
|  | Representativeness of exposed cohort | | Selection of the non-  exposed  cohort | Ascertainment of exposure | Demonstration that outcome of interest was not present at start of study | Comparability  of cohorts on the basis of the design or analysis | Assessment of outcome | Was follow-  up long enough for  outcomes to  occur | Adequacy  of follow  up of  cohorts |  |
| Hung Y. C (2017) | | 1 | 1 | 1 | 1 | 2 | 1 | 1 | 1 | 9 |
| Wallander M (2017) | | 1 | 1 | 1 | 1 | 2 | 1 | 1 | 1 | 9 |
| Colhoun (2012) | | 1 | 1 | 1 | 1 | 2 | 1 | 1 | 1 | 9 |
| Majumdar (2016) | | 1 | 1 | 1 | 1 | 2 | 1 | 1 | 1 | 9 |
| Tak Kyu Oh (2020) | | 1 | 1 | 1 | 1 | 0 | 1 | 1 | 1 | 7 |
| Starup-Linde J (2017) | | 1 | 1 | 1 | 1 | 2 | 1 | 1 | 1 | 9 |
| Tseng C-H (2021) | | 1 | 1 | 1 | 1 | 2 | 1 | 1 | 1 | 9 |

**Table S3** The Newcastle Ottawa scale for case-control study

| **Study** | **Selection** | | | | **Comparability** | **Exposure** | | | **Total stars** |
| --- | --- | --- | --- | --- | --- | --- | --- | --- | --- |
|  | Adequacy  of case  definition | Representativeness of the cases | Selection of controls | Definition of controls | Comparability  of cases and controls on the basis of the design or analysis | Ascertainment of exposure | Same method of ascertainment | Non-response rate |  |
| Vavanikunnel J  (2019) | 1 | 1 | 1 | 1 | 2 | 1 | 1 | 0 | 8 |
| Puar, T. H  (2012) | 1 | 1 | 1 | 1 | 2 | 1 | 1 | 0 | 8 |
| Charlier  (2021) | 1 | 1 | 1 | 1 | 2 | 1 | 1 | 0 | 8 |
| Monami M  (2008) | 1 | 1 | 1 | 1 | 2 | 1 | 1 | 0 | 8 |
